# In-hospital adverse outcomes and risk factors among chronic kidney disease patients infected with the omicron variant of SARS-CoV-2: a single-center retrospective study

**DOI:** 10.1186/s12879-023-08620-2

**Published:** 2023-10-18

**Authors:** Yue Guo, Yifei Guo, Huajian Ying, Weien Yu, Shiqi Chen, Yao Zhang, Shenyan Zhang, Yanxue Lin, Feng Sun, Yongmei Zhang, Jie Yu, Ke Ma, Lunxiu Qin, Feng Long, Haoxiang Zhu, Richeng Mao, Jun Xue, Jiming Zhang

**Affiliations:** 1 Department of Infectious Diseases, Shanghai Key Laboratory of Infectious Diseases and Biosafety Emergency Response, Shanghai Institute of Infectious Diseases and Biosecurity, Huashan Hospital, National Medical Center for Infectious Diseases, Fudan University, Shanghai, China; 2 Department of Nephrology, Huashan Hospital Fudan University, Shanghai, China; 3 Department of Emergency and Acute Critical Care, Huashan Hospital North, Fudan University, Shanghai, China; 4 Department of General Surgery, Cancer Metastasis Institute, Huashan Hospital, Fudan University, Shanghai, China; 5 Department of Respiratory Medicine, Huashan Hospital North, Fudan University, Shanghai, China; 6 Key Laboratory of Medical Molecular Virology (MOE/MOH), Shanghai Medical College, Fudan University, Shanghai, China; 7 Department of Infectious Diseases, Jing’An Branch of Huashan Hospital, Fudan University, Shanghai, China

**Keywords:** COVID-19, Omicron, Chronic kidney disease, Adverse outcome, Risk factor

## Abstract

**Introduction:**

The SARS-CoV-2 Omicron variant has decreased virulence and pathogenicity, yet the number of Omicron infections worldwide is unprecedentedly high, with rather high mortality and severe disease rate. Chronic kidney disease (CKD) patients are particularly vulnerable to the SARS-CoV-2 Omicron variant and have unique clinical outcomes.

**Methods:**

We retrospectively collected data from 2140 hospitalized patients with SARS-CoV-2 Omicron variant infection from March 29, 2022, to May 17, 2022. Demographic characteristics, ancillary examination results, and clinical treatments were described. Occurrence of critical COVID-19 or death and time of positive-to-negative conversion was defined as primary outcomes. The presence of COVID-19 pneumonia and the usage of respiratory or circulatory support was defined as secondary outcomes. Univariate or multivariate logistic regression analyses were performed to identify risk factors for primary outcomes.

**Results:**

15.74% of CKD patients infected with the SARS-CoV-2 Omicron variant ended up with critical COVID-19 or death. Pre-existing CKD was a risk factor for critical COVID-19 or death and prolonged time of positive-to-negative conversion of SARS-CoV-2. Nirmatrelvir-ritonavir facilitated viral clearance among COVID-19 patients with non-severe CKD.

**Conclusion:**

We found patients with CKD and COVID-19 due to Omicron experienced worse clinical outcomes and prolonged time of positive-to-negative conversion of SARS-CoV-2 compared to patients without CKD, which helps rationalize limited medical resources and offers guidance for appropriate clinical treatments.

**Supplementary Information:**

The online version contains supplementary material available at 10.1186/s12879-023-08620-2.

## Background

The SARS-CoV-2 Omicron variant, the recently occurred and dominating variant of concern (VOC), emerged from South Africa and Botswana in November 2021 and has been detected in at least 201 countries. Compared to previous SARS-CoV-2 variants, Omicron is less virulent and less pathogenic [1], with an increased proportion of asymptomatic and mild cases [2, 3]. However, the infection rate of Omicron was unprecedentedly high, and no significant decrease was found in the mortality rate and severe disease rate [4]. Due to a large number of severe and critical COVID-19 cases, there was a large shortage of medical equipment and healthcare workers, bringing great challenges to public health facilities, especially in underdeveloped countries [5].

Chronic kidney disease (CKD) occurs when the kidneys are damaged and cannot filter blood, causing excessive fluid and blood waste and added risk for heart disease, stroke, and so on. Data from the Lancet showed the prevalence of CKD varies from 8 to 16% worldwide, leading to 0.36 billion disability-adjusted life years globally in 2017 [6].Insights from epidemiology experts believed that patients receiving in-hospital dialysis had a higher risk for SARS- CoV-2 exposure compared to the general population, owing to the lack of quarantine conditions [7]. CKD patients with COVID-19 may have a higher incidence of death than CKD patients without COVID-19 [8].

Shanghai experienced a COVID-19 Omicron wave in March 2022, caused mainly by the BA.2 strain [9]. As one of the largest designated hospitals for COVID-19 patients, the Baoshan Branch of Huashan Hospital, Fudan University was equipped with various specialized departments and facilities for the treatment of patients with a higher risk of progression to critical illness and death. Our objective is to investigate the differences between COVID-19 patients with and without CKD regarding the emergence of adverse outcomes and time of positive-to-negative conversion during the Omicron wave.

## Methods

### Study design

This is a retrospective observational study initiated by Huashan Hospital, Fudan University (protocol number: KY2022-582). Informed consent forms were signed by each patient. Clinical data of COVID-19 patients from the electronic health records of the Baoshan Branch of Huashan Hospital were collected and analyzed. A total of 2140 hospitalized COVID-19 patients were consecutive selected from March 29 to May 17, 2022.

### Inclusion and exclusion criteria

#### Inclusion criteria

All real-time polymerase chain reaction (PCR)-confirmed COVID-19 patients hospitalized in the Baoshan Branch of Huashan Hospital were included for further screening.

#### Exclusion criteria

1. Patients < 18 years old;
2. Patients under pregnancy or lactation;
3. Patients with mental illness;
4. Patients without survival data. (The final follow-up date was June 17, 2022.)

#### Data collection

The following clinical data were collected: (1) Basic characteristics: age, gender, COVID-19 vaccination status, body mass index(BMI) and comorbidity (chronic kidney disease, hypertension, diabetes mellitus, cardiovascular disease, malignancy, neurological disease, chronic lung disease, chronic liver disease, rheumatic disease, hemopathy); (2) Results of first laboratory and auxiliary examinations after hospital admission: white blood cells (WBC), lymphocyte count, hemoglobin(Hb), blood platelet(PLT), lactic acid(LAC), C-reactive protein (CRP), procalcitonin (PCT), D-dimer, cardiac troponin T(cTnT), brain natriuretic peptide(BNP), serum creatinine(Cr), estimated glomerular filtration rate (eGFR), lactate dehydrogenase (LDH), SARS-CoV-2 ORF1b gene, N gene, and chest computed tomography; (3) COVID-19 treatment; (4) Clinical outcome: time of positive-to-negative conversion of SARS-CoV-2; critical COVID-19 and death; confirmed COVID-19 pneumonia by CT; oxygen face mask; high-flow nasal oxygen therapy; non-invasive ventilation; mechanical ventilation and vasopressors.

According to WHO definitions of disease severity(non-severe, severe, and critical) for COVID-19 [10], a critical COVID-19 case is defined by the criteria for acute respiratory distress syndrome (ARDS), sepsis, septic shock, or other conditions that would normally require the provision of life-sustaining therapies such as mechanical ventilation (invasive or non-invasive) or vasopressor therapy.

Patients with no persistent CKD were classified as non-CKD. Patients with persistent CKD were classified as CKD. The CKD group was further classified into 5 subgroups according to the eGFR levels of patients (CKD-1, eGFR > 90; CKD-2, eGFR 60–90; CKD-3, eGFR 30–60; CKD-4, eGFR15-30; CKD-5, eGFR < 15).

Vaccination status was classified into 3 groups, including “unvaccinated”, “primary vaccination”, and “boostered”, where “unvaccinated” was defined as having not received any COVID-19 vaccine before infection, “primary vaccination” was defined as having received one or two doses of COVID-19 vaccines for at least 14 days; “boostered” was defined as having received three or four doses of COVID-19 vaccines for at least 14 days.

### Outcomes

Primary outcomes include (1) Critical COVID-19 and death, (2) Time of positive-to-negative conversion of SARS-CoV-2. Secondary outcomes include (1) CT-confirmed pneumonia, and (2) Need for any form of respiratory support (oxygen face mask, high-flow oxygen therapy or non-invasive ventilation, mechanical ventilation, or vasopressors). Since they are not mutually exclusive, a patient may experience more than one outcome during hospitalization.

### Statistical analysis

Mean ± standard deviation or median with an interquartile range was used to describe quantitative data. The normality of the data was assessed by the Kolmogorov-Smirnov test and the Shapiro-Wilk test. The chi-square test or Fisher’s exact probability test was used to compare enumeration data between groups with constituent ratios and rates (percentages). Data conforming to normal distribution and homogeneity of variance were compared using the independent sample T-test. Data with skewed distribution were compared using the Mann-Whitney U test. Multivariable models were built through backward stepwise selection, with a p-value less than 0.05 required for inclusion. Propensity score matching at 1:1 ratio was conducted before logistic regression analysis to match CKD and non-CKD groups. Chronic kidney disease, diabetes mellitus, cardiovascular disease, malignancy, neurological disease was included in the multivariable model to assess their association with critical COVID-19 and death outcome. Kaplan-Meier method and log-rank method were used to compare the time of positive-to-negative conversion of SARS-CoV-2 between groups.

SPSS 26.0 software was used for data analysis. GraphPad Prism 8.0 software and R. 4.2.1 were used to draw charts. A p-value of less than 0.05 was considered statistically significant.

## Results

### Characteristics of study participants

A total of 2140 COVID-19 patients were hospitalized in the Baoshan Branch of Huashan Hospital from March 30 to May 17, 2022. After screening according to the inclusion and exclusion criteria, 1978 patients remained and were finally included in the study. There were 470/1978 (23.76%) patients with CKD and 1508/1978 (76.24%) patients without CKD. In the CKD group, 206/470 (43.83%) individuals received hemodialysis treatment during hospitalization (Supplementary material Fig. 1).

The percentage of males in the CKD group was 56.60%. The median age was 73 years old, of which 48.30% were 60–80 years old and 35.53% were over 80 years old. The vaccination rate was 12.10%. The percentage of males in the non-CKD group was 45.36%. The median age was 62 years, of which 43.04% were 60–80 years old and 11.67% were over 80 years old. The vaccination rate was 60.00%. Most patients of the CKD group had a variety of underlying diseases, including hypertension, diabetes, heart disease, neurological disease, and malignancy (Table 1). Supplementary material Fig. 2 illustrates the specific comorbidities.

**Table 1:** Demographic, laboratory examination results, and treatment of hospitalized COVID-19 patients on admission

	Level	Non-CKD group (n = 1508)	CKD group(n = 470)	P-value
Age(years), median [Q1-Q3]		62 [47–71]	73 [64–86]	< 0.001*
Age group (%)	18 ~ 40	269 (17.84)	16 (3.40)	< 0.001*
	40 ~ 60	414 (27.45)	60 (12.77)	
	60 ~ 80	649 (43.04)	227 (48.30)	
	80+	176 (11.67)	167 (35.53)	
Gender (%)	Female	824 (54.64)	204 (43.40)	< 0.001*
	Male	684 (45.36)	266 (56.60)	
Body mass index, BMI (kg/cm^2^), median [Q1-Q3]		23.15 [20.83–25.39]	22.49 [20.73–24.99]	0.496
Vaccination status (%)	Unvaccinated	532 (35.28)	391 (83.19)	< 0.001*
	Primary vaccination	442(29.31)	34(7.23)	
	Booster	463 (30.70)	23 (4.89)	
	Unclear	71 (4.71)	22 (4.68)	
Hypertension (%)	No	768 (50.93)	116 (24.68)	< 0.001*
	Yes	740 (49.07)	354 (75.32)	
Diabetes mellitus (%)	No	1268 (84.08)	299 (63.62)	< 0.001*
	Yes	240 (15.92)	171 (36.38)	
Cardiovascular disease (%)	No	1355 (89.85)	363 (77.23)	< 0.001*
	Yes	153 (10.15)	107 (22.77)	
Malignancy (%)	No	1404 (93.10)	447 (95.11)	0.1501
	Yes	104 (6.90)	23 (4.89)	
Neurological disease (%)	No	1368 (90.72)	423 (90.00)	0.7091
	Yes	140 (9.28)	47 (10.00)	
Chronic lung disease (%)	No	1464 (97.08)	461 (98.09)	0.3115
	Yes	44 (2.92)	9 (1.91)	
Chronic liver disease (%)	No	1467 (97.28)	458 (97.45)	0.9756
	Yes	41 (2.72)	12 (2.55)	
Rheumatic disease (%)	No	1491 (98.87)	465 (98.94)	1
	Yes	17 (1.13)	5 (1.06)	
Hemopathy (%)	No	1491 (98.87)	462 (98.30)	0.4608
	Yes	17 (1.13)	8 (1.70)	
Number of other comorbidities (%)	0	558 (37.00)	65 (13.83)	< 0.001*
	1	564 (37.40)	180 (38.30)	
	2	256 (16.98)	141 (30.00)	
	≥ 3	130 (8.62)	84 (17.87)	
Laboratory findings				
White blood cells (%)	> 10*10^9/L	70 /1131(6.19)	42/460 (9.13)	0.002*
	< 4*10^9/L	274/1131 (24.23)	138/460 (30.00)	
Lymphocyte (%)	< 0·8*10^9/L	183/1128 (16.22)	173/460 (37.61)	< 0.001*
Hemoglobin (%)	< 90 g/l	33/1131 (2.92)	68/460 (14.78)	< 0.001*
Platelets (%)	> 300*10^9/L	98/1131 (8.66)	21/460 (4.57)	< 0.001*
	< 100*10^9/L	50/1131 (4.42)	70/460 (15.22)	
Lactic acid (%)	> 2.45mmol/ L	42/323 (13.00)	16/97 (16.49)	< 0.001*
C-reactive protein (%)	> 10 mg/L	529/674 (78.49)	146/197 (74.11)	< 0.001*
Procalcitonin (%)	> 0.5ng/ml	112/488 (22.95)	43/163 (26.38)	< 0.001*
D-dimer (%)	> 1ug/ml	333/1081 (30.80)	96/343 (27.99)	< 0.001*
Troponin T (%)	> 0.5 µg/L	11/1017 (1.07)	3/331 (0.91)	< 0.001*
B-type natriuretic peptide (%)	> 450pg/ml	314/1021 (30.75)	90/337 (27.61)	< 0.001*
Lactate dehydrogenase (%)	> 300U/L	76/872 (8.72)	28/283 (9.89)	< 0.001*
Estimated glomerular filtration rate (%)	> 90	549 (48.80)	3 (0.65)	< 0.001*
	61 ~ 90	566 (50.31)	7 (1.52)	
	31 ~ 60	5 (0.44)	197 (42.83)	
	< 30	5 (0.44)	253 (55.00)	
Alanine aminotransferase (%)	> 40u/l	97/1111 (8.73)	47/438 (10.73)	0.2612
Aspartate aminotransferase (%)	> 40u/l	86/1116 (7.71)	61/452 (13.50)	< 0.001*
PCR-Ct, median (Q1-Q3)				
N-gene		23.490 [19.040-32.300]	21.585 [18.387-28.808]	< 0.001*
ORF1ab-gene		26.785 [22.530-34.952]	24.325 [21.428-31.495]	< 0.001*
COVID-19 treatment				
Hemodialysis (%)	Yes		206(43.83)	< 0.001*
Paxlovid (%)	No	896 (59.42)	303 (64.47)	0.057
	Yes	612 (40.58)	167 (35.53)	
Heparin (%)	No	1257 (83.36)	187 (39.79)	< 0.001*
	Yes	251 (16.64)	283 (60.21)	
Glucocorticoids (%)	No	1445 (95.82)	408 (86.81)	< 0.001*
	Yes	63 (4.18)	62 (13.19)	

Data are median [Q1-Q3], n (%), or n/N (%). p values were calculated by the Mann-Whitney U test, χ² test, or Fisher’s exact test, as appropriate. *Statistically significant

Hospitalized COVID-19 patients: Patient hospitalized for Coronavirus disease 2019 during Omicron wave. Non-CKD group: Patients without chronic kidney disease. CKD group: Patients with chronic kidney disease. BMI: Body mass index. Vaccination status: Unvaccinated: No vaccination; Primary vaccination: One or two doses of vaccination; Booster: Three doses of vaccination; Unclear: Not documented in medical records. Number of other comorbidities: Number of comorbidities other than chronic kidney disease. PCR-Ct: Cycle threshold of N and ORF1ab genes for novel coronavirus nucleic acid detection by Reverse transcription polymerase chain reaction. Glucocorticoids: the application of dexamethasone and/or methylprednisolone treatment

Post-admission laboratory tests showed that patients with CKD were more likely to have leukocytosis (CKD 9.13% vs. non-CKD 6.19%) and leukopenia (CKD 30.00% vs. non-CKD 24.23%); a higher proportion of patients with lymphopenia (CKD 37.61% vs. non-CKD 16.22%); anemia (CKD 14.78% vs. non-CKD 2.92%); thrombocytopenia (CKD 15.22% vs. non-CKD 4.42%)and other abnormalities (elevated lactate PCT, lactate dehydrogenase, AST). The median of cycle threshold (Ct) values of the CKD group was lower than that of the non-CKD group (N gene, 21.585 for CKD and 23.490 for non-CKD; ORF1ab gene, 24.325 for CKD and 26.785 for non-CKD; *p* < 0.001)(Table 1).

No significant difference in nirmatrelvir/ritonavir (Paxlovid) treatment was observed between the two groups (35.53% for CKD and 40.58% for non-CKD, *p* = 0.058). Heparin and glucocorticoids were more commonly used on patients in the CKD group (Heparin 283/470, 60.21%; Glucocorticoids 62/470, 13.19%) than in the non-CKD group (Heparin 251/1508, 16.64%; Glucocorticoids 63/1508, 4.18%) (*p* < 0.001) (Table 1).

### Incidence of adverse outcomes

**Fig. 1:**
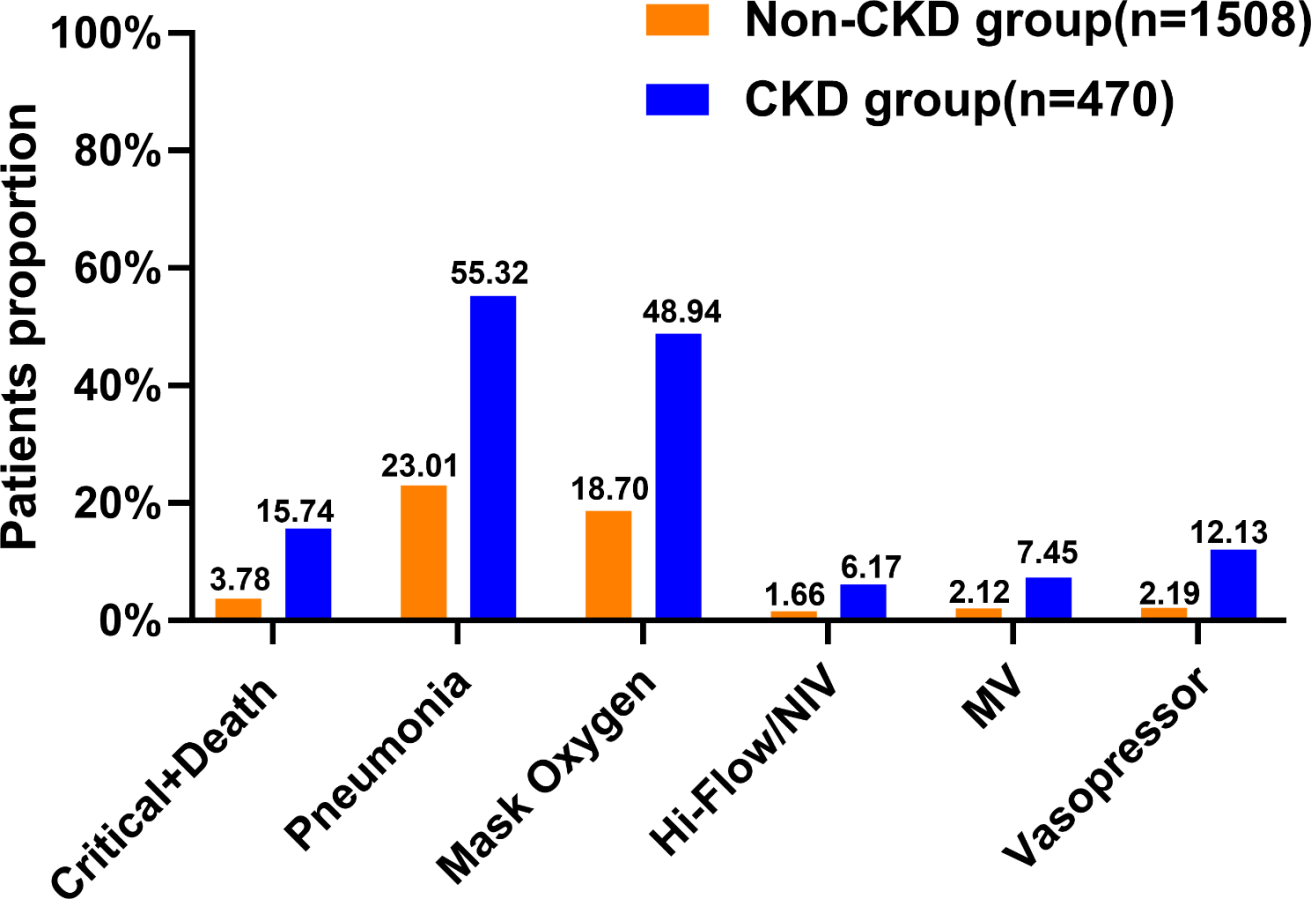
Comparison of six adverse clinical outcomes in COVID-19 patients.The percentage of the total number of patients in each group with the above outcomes was higher in the CKD group than in the non-CKD group, and the difference was statistically significant (p < 0.001). Orange represents COVID-19 patients without CKD and blue represents COVID-19 patients with pre-existing CKD.NIV: Non-invasive ventilator; MV: Mechanical Ventilation

Patients with CKD had a higher rate of adverse outcomes. Figure 1 showed that the incidence of critical COVID-19 and death was higher in the CKD group than in the non-CKD group (15.74% in CKD vs. 3.78% in non-CKD, p < 0.001). Higher incidence of pneumonia in the CKD group compared to the non-CKD group (55.32% in CKD vs. 23.01% in non-CKD, p < 0.001). Respiratory and circulatory support treatments were more frequently used in the CKD group, including mask oxygenation (48.94% in CKD vs. 18.70% in non-CKD, p < 0.001), high-flow oxygen therapy or non-invasive ventilation (6.17% in CKD vs. 1.66% in non-CKD, p < 0.001), mechanical ventilation (7.45% vs. 2.12% in non-CKD, p < 0.001) and vasopressors (12.13% in CKD vs. 2.19% in non-CKD, p < 0.001).

Subgroup analysis according to age group showed that incidence of critical COVID-19 and death, pneumonia, mask oxygenation, high-flow oxygenation, and mechanical ventilation were significantly higher in the CKD group than in the non-CKD group, with statistically significant differences (except for the 18–40 years group, where there were no patients with mechanical ventilation and vasopressor). In patients over 80 years of age, there were no significant differences in the six outcomes described above (Supplementary material Fig. 3).

### Risk factors for critical COVID-19 and death among COVID-19 patients

Considering the less pathogenicity [11] and the lower in-hospital mortality caused by the Omicron variant [12, 13] compared to early SARS-CoV-2 strains, we used critical COVID-19 and in-hospital death as our primary outcome. Chronic kidney disease was associated with primary outcome in supplement Table 1 and supplement Table 2. Multivariable logistic regression analyses showed that chronic kidney disease (adjusted OR = 3.69, 95% CI: 2.09–6.25), diabetes mellitus (adjusted OR = 2.20, 95% CI: 1.40–3.47), cardiovascular disease (adjusted OR = 4.00, 95% CI: 2.53–6.32), malignancy adjusted OR = 3.96, 95% CI: 1.91–8.22), and neurological disease (adjusted OR = 5.07, 95% CI: 2.93–8.76) were the independent risk factors for critical COVID-19 and in-hospital death in Omicron COVID-19 patients after propensity score matching.

**Table 2:** Risk factors of Critical COVID-19 and Death outcome among CKD patients

	Univariate			Multivariate		
	OR	CI	P	Adjusted OR	CI	P
Gender						
Female	ref	ref	ref			
Male	3.72	1.06–13.13	0.04*			
Vaccination status						
No	ref	ref	ref			
Yes	0.96	0.21–4.33	0.96			
Hypertension						
No	ref	ref	ref			
Yes	1.07	0.34–3.34	0.91			
Diabetes mellitus						
No	ref	ref	ref			
Yes	3.36	1.22–9.25	0.02*			
Cardiovascular disease						
No	ref	ref	ref	ref	ref	ref
Yes	6.82	2.46–18.9	< 0.001*	2.45	1.41–4.25	< 0.001*
Malignancy						
No	ref	ref	ref	ref	ref	ref
Yes	7.03	2.09–23.6	< 0.001*	5.10	2.94–8.85	< 0.001*
Neurological disease						
No	ref	ref	ref	ref	ref	ref
Yes	1.21	0.27–5.46	0.81	5.30	2.02–13.85	< 0.001*
Chronic lung disease						
No	ref	ref	ref			
Yes	0	0-Inf	0.99			
Chronic liver disease						
No	ref	ref	ref			
Yes	2.51	0.31–20.65	0.39			
Rheumatic disease						
No	ref	ref	ref			
Yes	0	0-Inf	0.99			
Hemopathy						
No	ref	ref	ref			
Yes	1.94	0.57–6.58	0.29			
CKD stage						
1 ~ 3	ref	ref	ref			
4 ~ 5	2.17	0.75–6.25	0.15			
Number of other comorbidities						
0	ref	ref	ref			
1	3,532,202	0-Inf	0.99			
2	13,971,823	0-Inf	0.99			
≥ 3	37,723,921	0-Inf	0.99			
Age group						
18 ~ 40y	ref	ref	ref			
40 ~ 60y	4,057,852	0-Inf	0.99			
60 ~ 80y	5,329,438	0-Inf	0.99			
> 80y	3,636,755	0-Inf	0.99			

Bivariable and multivariable logistic regression was used. Multivariable logistic regression adjusted for gender, age group, vaccination status, diabetes mellitus, cardiovascular disease, malignancy, neurological disease, chronic liver disease and CKD stage. Only statistically significant values are given for the adjusted OR. *Statistically significant

Table 2 showed that CKD patients with cardiovascular disease, malignancy, and neurological disease were more likely to have critical COVID-19 and death than those without these diseases (cardiovascular disease: adjusted OR = 2.45, 95% CI:1.41–4.25, malignancy: adjusted OR = 5.10 , 95% CI:2.94–8.85; and neurological disease: adjusted OR = 5.20, 95% CI:2.02–13.85).

### Time of positive-to-negative conversion of SARS-CoV-2

**Fig. 2:**
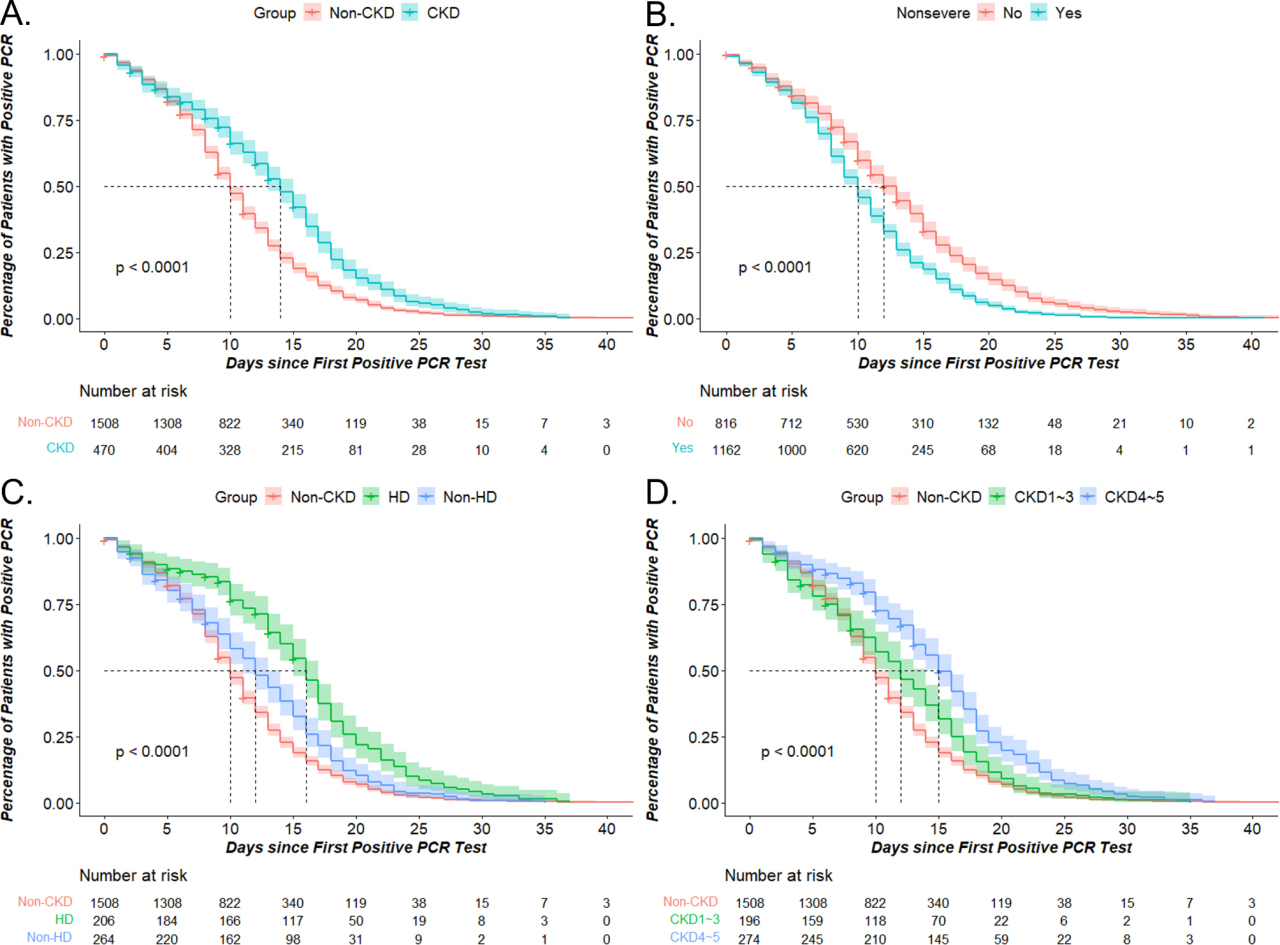
The time of positive-to-negative conversion of SARS-CoV-2 in COVID-19 patients. The KM of time of positive-to-negative conversion of SARS-CoV-2 according to CKD **(A)**, severity **(B)**, hemodialysis **(C)**, CKD stage **(D)**. Nonsevere: Yes = non-severe group; No = severe and critical group

**Fig. 3:**
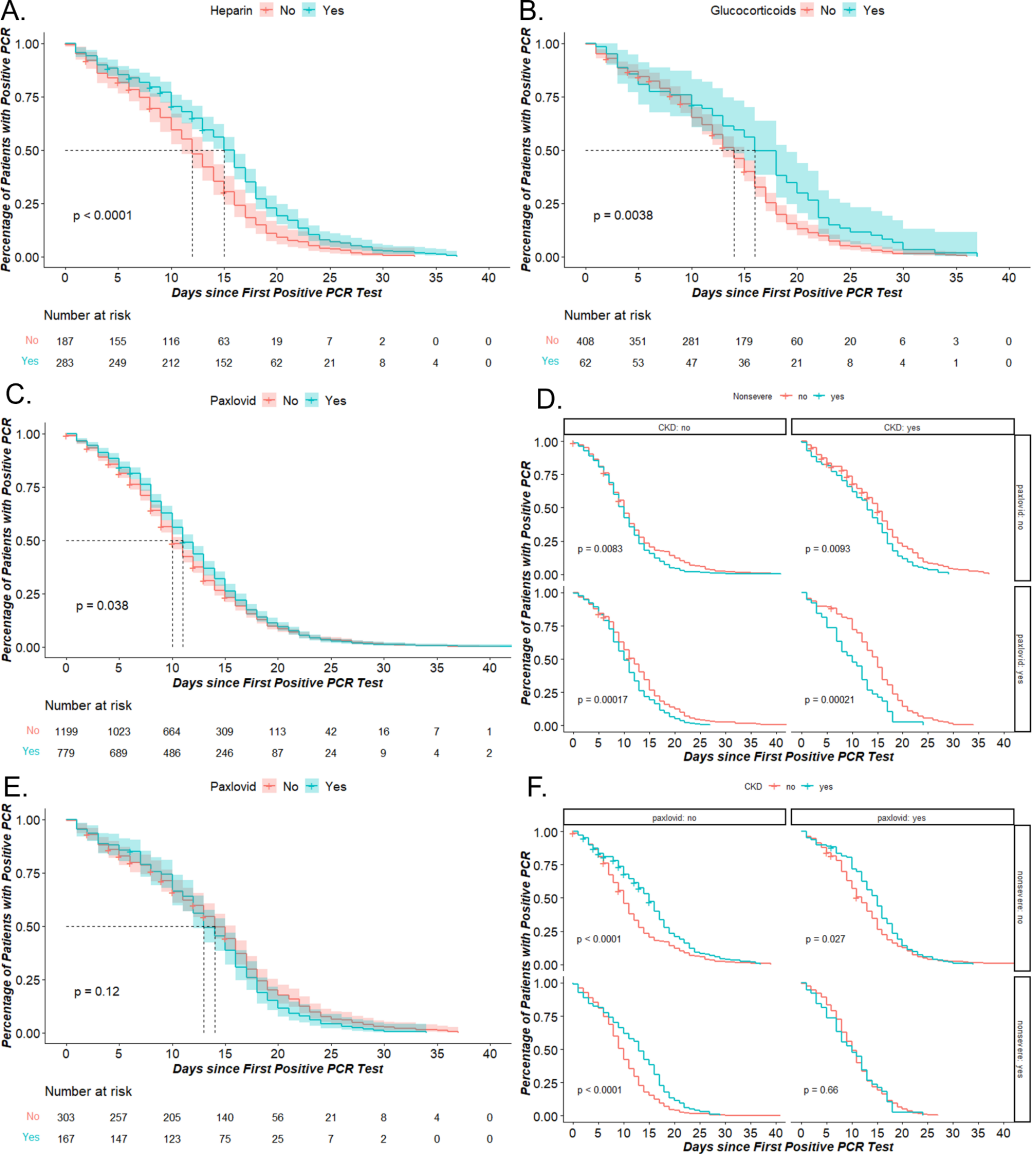
The time of positive-to-negative conversion of SARS-CoV-2 according drugs in COVID-19 patients. The KM of time of positive-to-negative conversion of SARS-CoV-2 according to heparin **(A)**, glucocorticoids **(B)**, paxlovid **(C)** in COVID-19 patients. The KM of time of positive-to-negative conversion of SARS-CoV-2 between non-severe and severe group according to paxlovid and CKD **(D)**, and between CKD and non-CKD according to paxlovid and severity **(F)**. The KM of time of positive-to-negative conversion of SARS-CoV-2 in CKD group according to paxlovid **(E)**. Nonsevere: yes = non-severe group; no = evere and critical group. **(E)** and **(F)** *p < 0.0125 Bonferroni correction

The median time of positive-to-negative conversion of SARS-CoV-2 was prolonged in the CKD group compared to the non-CKD group (14 vs. 10 days) as shown in Fig. 2A. Figure 2B demonstrated that the severe group had a longer median time of positive-to-negative conversion than the non-severe group. CKD stage, according to eGFR levels during hospitalization and maintenance hemodialysis or peritoneal dialysis status, were also influential factors on time of positive-to-negative conversion of SARS-CoV-2. Patients with CKD stage 4–5 had a longer time of positive-to-negative conversion than those with stages 1–3, indicating that CKD stage is an important factor in prolonging viral clearance for CKD patients (Fig. 2C). Additionally, among different treatment groups, the hemodialysis group had a significantly longer median time of positive-to-negative conversion than others (HD:16 days vs. Non-HD:12 days vs. Non-CKD:10 days) as shown in Fig. 2D. Furthermore, drugs such as heparin and glucocorticoids were found to be associated with prolonged viral clearance times (Fig. 3A and 3B). Figure 3C and 3E depicted the effects of nirmatrelvir/ritonavir (Paxlovid) on COVID-19 patients and specifically on CKD COVID-19 patients, respectively. Additionally, we observed that non-severe CKD COVID-19 patients who were administered this drug experienced an earlier time of positive-to-negative conversion which suggests that nirmatrelvir/ritonavir may facilitate viral clearance in non-severe cases among CKD patients (Fig. 3D and 3F).

## Discussion

In this retrospective study among COVID-19 patients, we observed the incidence of adverse outcomes during the Omicron wave in Shanghai. Our results demonstrated that patients with COVID-19 and CKD had an increased risk of adverse outcomes, such as higher critical COVID-19 and death rate and higher pneumonia rate. Pre-existing CKD was a significant risk factor for the prolonged time of positive-to-negative conversion of SARS-CoV-2 and the development of critical COVID-19 and death.

Patients with pre-existing CKD were the vulnerable population for Omicron infection. The Open SAFELY study [14] found that CKD (eGFR < 30 mL/ minute /1.73 m^2^) was the risk factor for death among 17 million COVID-19 patients before the emergence of Omicron. STOP-COVID [15]study detected higher mortality rates for both dialysis-dependent and -independent CKD patients. A multi-center study had the same conclusion as this study [16].Our study found 15.74% of patients in the CKD group developed critical COVID-19 and death. It was lower than the case fatality rate of 50% in the study conducted in the United States [15]. The risk factors for the development of critical COVID-19 and in-hospital death for CKD patients included cardiac disease, malignancy, and neurological disease, which had been known among patients suffering from CKD [15].

The quantitative assessment of viral RNA through RT-PCR is a highly sensitive approach for evaluating the extent and duration of infectious virus replication, which plays a crucial role in assessing transmission risk and guiding decisions regarding patient isolation [17].Age > 60 years [18], comorbidities (HIV infection, diabetes mellitus, hemodialysis treatment) [19–22]or transplant status [23] can lead to long-term viral shedding among those who are immunocompromised or receiving immunosuppressive therapy [24].Time of viral shedding was prolonged in the CKD population compared to other individuals [25]. CKD patient’s immune system was different from that of other individuals.Unfortunately, the exact mechanism remains unclear and may be related to adaptive immune deficiency [26], including less active T-cell responses at the initial phase of infection [27], B-cell count decreases [28]and abnormal proinflammatory cytokines [29].

Additionally, drug intervention is a crucial factor influencing the prolonged shedding duration of SARS-CoV-2 RNA. Glucocorticoids and heparin have been shown to prolong SARS-CoV-2 RNA shedding time [30], while antiviral drugs have a positive effect on shortening it. Numerous studies have demonstrated that Paxlovid can effectively reduce Omicron variant viral loads and shorten polymerase chain reaction time of positive-to-negative conversion [31–33]. Furthermore, it has proven effective in special populations such as elderly hospitalized patients [34], immunocompromised patients [35], kidney transplantation [36, 37]. Cai et al. found the viral load decreased faster in patients with eGFR < 90 ml/min who received Paxlovid treatment ≤ 5 days [38].Our results indicate that non-severe CKD COVID-19 patients are optimal candidates. Selecting appropriate populations and timing treatment may be more beneficial for viral clearance.

There are some limitations in our study. Firstly, this is a single-center retrospective study with limited participants and laboratory results. Prospective cohort studies can be conducted to control the bias in the future. Secondly, we recognise clinical outcomes (death and critical COVID-19) as composite outcome due to low incidence rates, this limitation restricts the interpretation of the study. Thirdly, the CKD group was defined based on hospital electronic medical record data, which may be subject to recall bias. Mandatory albuminuria testing should be considered for all individuals who test positive for COVID-19 and are hospitalized, which is beneficial to distinguish the different stages of CKD. Last but not least, we have taken a first glimpse at the effectiveness of nirmatrelvir/ritonavir in patients with CKD. Prospective controlled trials should be designed to assess the safety, efficacy, and optimal timing of nirmatrelvir/ritonavir usage in the future.

## Conclusions

In conclusion, we found patients with CKD and COVID-19 due to Omicron experienced worse clinical outcomes and prolonged time of positive-to-negative conversion of SARS-CoV-2 compared to patients without CKD, which will help rationalize the limited medical resources and help patients receive appropriate treatment.

### Electronic supplementary material

Below is the link to the electronic supplementary material.

Supplementary Material 1

## Data Availability

The datasets used and/or analysed during the current study available from the corresponding author on reasonable request.

## Abbreviations

CKD: Chronic kidney disease
HD: Hemodialysis
COVID-19: Coronavirus disease 2019
PCR-Ct: Cycle Threshold during reverse transcription polymerase chain reaction (RT-PCR) assay
