# Supplementary material for: In-hospital adverse outcomes and risk factors among chronic kidney disease patients infected with the omicron variant of SARS-CoV-2: a single-center retrospective study
Source: BMC Infect Dis. 2023 Oct 18;23:698. doi: 10.1186/s12879-023-08620-2 (PMC10585898; doi:10.1186/s12879-023-08620-2)

Supplementary Table 1. Baseline after propensity score matching cohort for multivariable logistic analysis.

|  |  | before propensity score matching | | | after propensity score matching | | |
| --- | --- | --- | --- | --- | --- | --- | --- |
|  | level | Non-CKD group | CKD group | p | Non-CKD group | CKD group | p |
| n |  | 1508 | 470 |  | 433 | 433 |  |
| Age, median [Q1-Q3] |  | 62 [47-71] | 73 [64-86] | <0.001* | 72[65-83] | 72[64-84] | 0.6574 |
| Gender (%) | female | 824 (54.64) | 204 (43.40) | <0.001* | 203 (46.88) | 194 (44.80) | 0.585 |
|  | male | 684 (45.36) | 266 (56.60) |  | 230 (53.12) | 239 (55.20) |  |
| Vaccination status (%) | unvaccinated+unclear | 603 (39.99) | 413 (87.87) | <0.001* | 375 (86.61) | 376 (86.84) | 1.000 |
|  | vaccinated | 905 (60.01) | 57 (12.13) |  | 58 (13.39) | 57 (13.16) |  |
| Hypertension (%) | no | 768 (50.93) | 116 (24.68) | <0.001* | 114 (26.33) | 116 (26.79) | 0.939 |
|  | yes | 740 (49.07) | 354 (75.32) |  | 319 (73.67) | 317 (73.21) |  |
| Diabetes mellitus (%) | no | 1268 (84.08) | 299 (63.62) | <0.001* | 291 (67.21) | 281 (64.90) | 0.518 |
|  | yes | 240 (15.92) | 171 (36.38) |  | 142 (32.79) | 152 (35.10) |  |
| Cardiovascular disease (%) | no | 1355 (89.85) | 363 (77.23) | <0.001* | 339 (78.29) | 341 (78.75) | 0.934 |
|  | yes | 153 (10.15) | 107 (22.77) |  | 94 (21.71) | 92 (21.25) |  |
| Number of other comorbidities (%) | 0 | 558 (37.00) | 65 (13.83) | <0.001* | 51 (11.78) | 65 (15.01) | 0.057 |
|  | 1 | 564 (37.40) | 180 (38.30) |  | 148 (34.18) | 167 (38.57) |  |
|  | 2 | 256 (16.98) | 141 (30.00) |  | 135 (31.18) | 130 (30.02) |  |
|  | ≥3 | 130 (8.62) | 84 (17.87) |  | 99 (22.86) | 71 (16.40) |  |

Supplementary Table 2. Risk factors of Critical COVID-19 and Death outcome among COVID-19 patients.

|  | Unadjusted | | | Adjusted | | |
| --- | --- | --- | --- | --- | --- | --- |
|  | OR | CI | P | OR | CI | P |
| Age group | |  |  |  |  |  |
| 18~40y | ref | ref | ref |  |  |  |
| 40~60y | 2.12 | 0.25-17.74 | 0.487 |  |  |  |
| 60~80y | 2.51 | 0.33-18.96 | 0.374 |  |  |  |
| >80y | 6.27 | 0.83-47.22 | 0.075 |  |  |  |
| Gender |  |  |  |  |  |  |
| female | ref | ref | ref |  |  |  |
| male | 1.59 | 1.04-2.44 | 0.032* |  |  |  |
| Vaccination status | |  |  |  |  |  |
| no | ref | ref | ref |  |  |  |
| yes | 0.85 | 0.45-1.60 | 0.604 |  |  |  |
| Chronic kidney disease | |  |  |  |  |  |
| no | ref | ref | ref | ref | ref | ref |
| yes | 1.75 | 1.15-2.67 | 0.009* | 3.69 | 2.09-6.52 | 0.000* |
| Hypertension | |  |  |  |  |  |
| no | ref | ref | ref |  |  |  |
| yes | 1.22 | 0.75-1.98 | 0.426 |  |  |  |
| Diabetes mellitus | |  |  |  |  |  |
| no | ref | ref | ref | ref | ref | ref |
| yes | 2.512 | 1.657-3.81 | 0.000* | 2.20 | 1.40-3.47 | 0.001* |
| Cardiovascular disease | |  |  |  |  |  |
| no | ref | ref | ref | ref | ref | ref |
| yes | 5.02 | 3.27-7.71 | 0.000* | 4.00 | 2.53-6.32 | 0.000* |
| Malignancy |  |  |  |  |  |  |
| no | ref | ref | ref | ref | ref | ref |
| yes | 1.91 | 1.03-3.57 | 0.042* | 3.96 | 1.91-8.22 | 0.000* |
| Neurological disease | |  |  |  |  |  |
| no | ref | ref | ref | ref | ref | ref |
| yes | 3.804 | 2.41-6.00 | 0.000* | 5.07 | 2.93-8.76 | 0.000* |
| Chronic lung disease | |  |  |  |  |  |
| no | ref | ref | ref |  |  |  |
| yes | 0.78 | 0.18-3.38 | 0.735 |  |  |  |
| Chronic liver disease | |  |  |  |  |  |
| no | ref | ref | ref |  |  |  |
| yes | 1.18 | 0.34-4.04 | 0.798 |  |  |  |
| Rheumatic disease | |  |  |  |  |  |
| no | ref | ref | ref |  |  |  |
| yes | 0.67 | 0.09-5.25 | 0.703 |  |  |  |
| Hemopathy |  |  |  |  |  |  |
| no | ref | ref | ref |  |  |  |
| yes | 1.06 | 0.24-4.73 | 0.940 |  |  |  |
| Number of other comorbidities | | |  |  |  |  |
| 0 | ref | ref | ref |  |  |  |
| 1 | 1.21 | 0.39-3.77 | 0.748 |  |  |  |
| 2 | 11.34 | 3.96-32.44 | 0.000* |  |  |  |
| ≥3 | 4.54 | 1.58-13.06 | 0.005* |  |  |  |

Bivariable and multivariable logistic regression was used. Only statistically significant values are given for the adjusted OR. *Statistically significant.

Supplementary Figure 1. Selection of study participants during the SARS-CoV-2 Omicron wave.


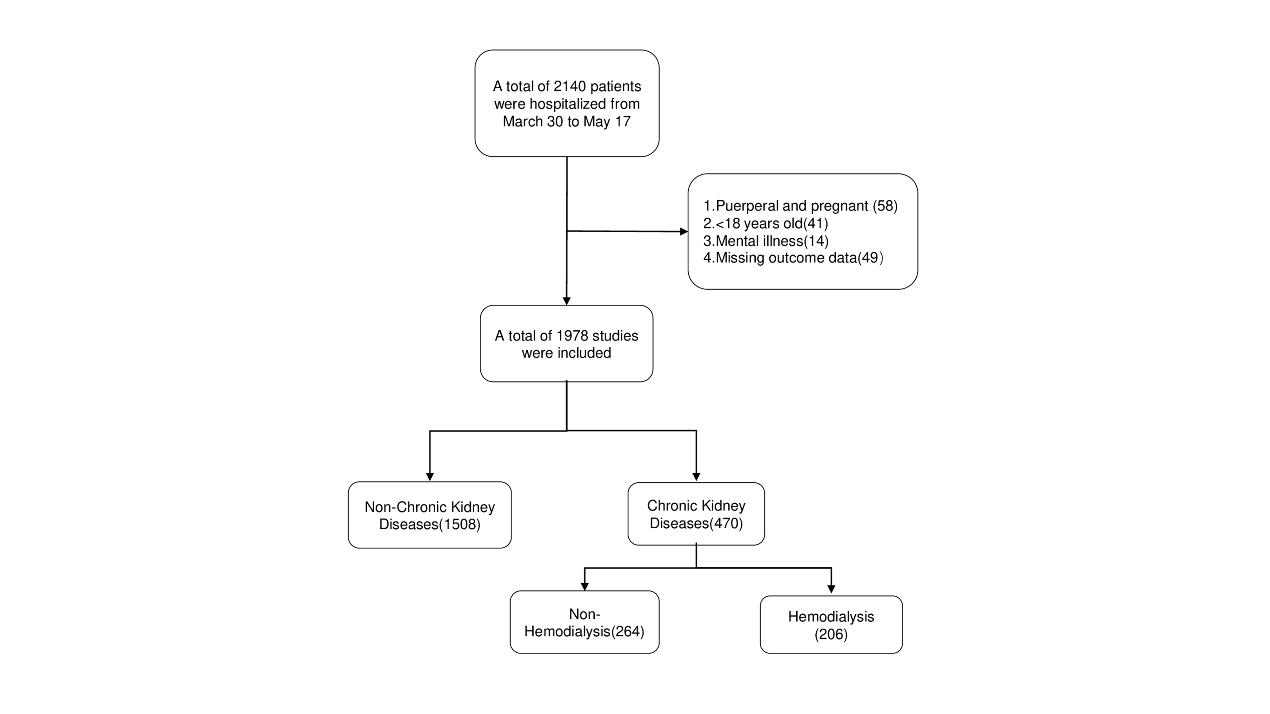


Supplementary Figure 2. Comorbidity specifics of COVID-19 patients in the CKD group.


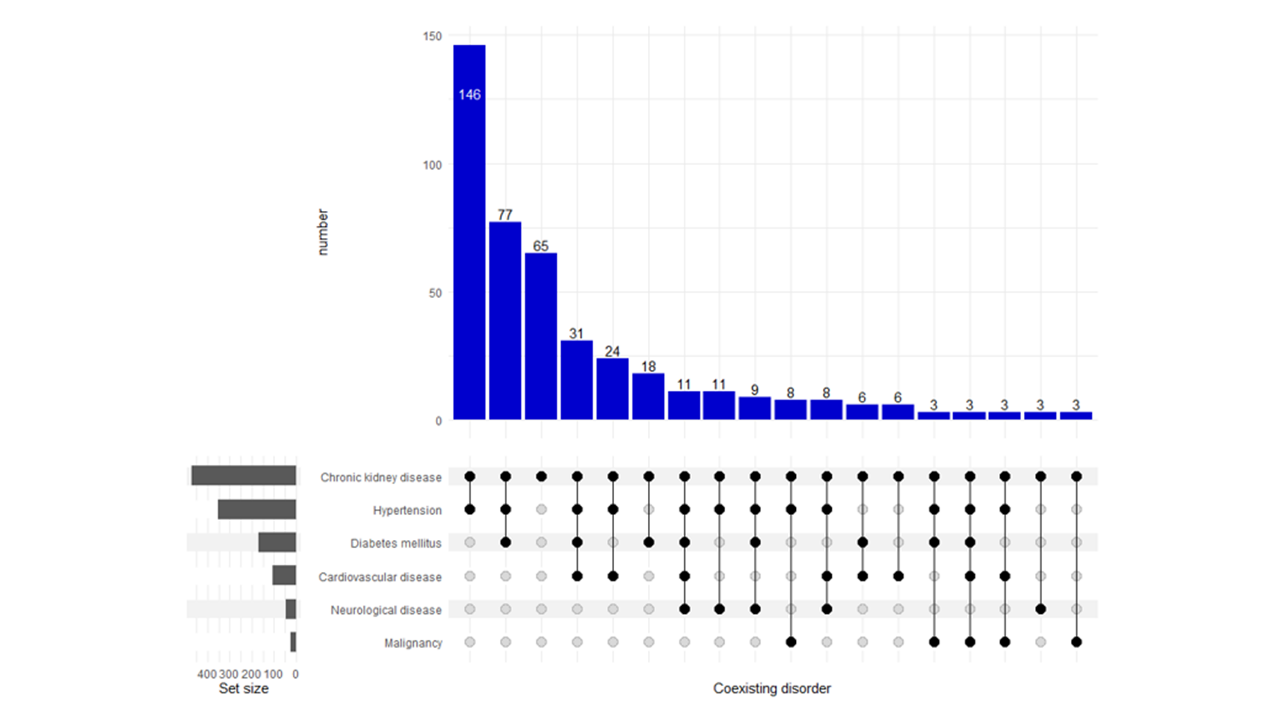


Supplementary Figure 3. Comparison of six adverse outcomes in age subgroups. A. The percent of critical COVID-19 and death in age subgroups. B. The percent of confirmed COVID-19 pneumonia in age subgroups. C. The use of vasopressor in age subgroups. D. The use of face mask oxygen in age subgroups. E. The use of high-flow nasal or non-invasive ventilation in age subgroups. F. The use of mechanical ventilation in age subgroups.


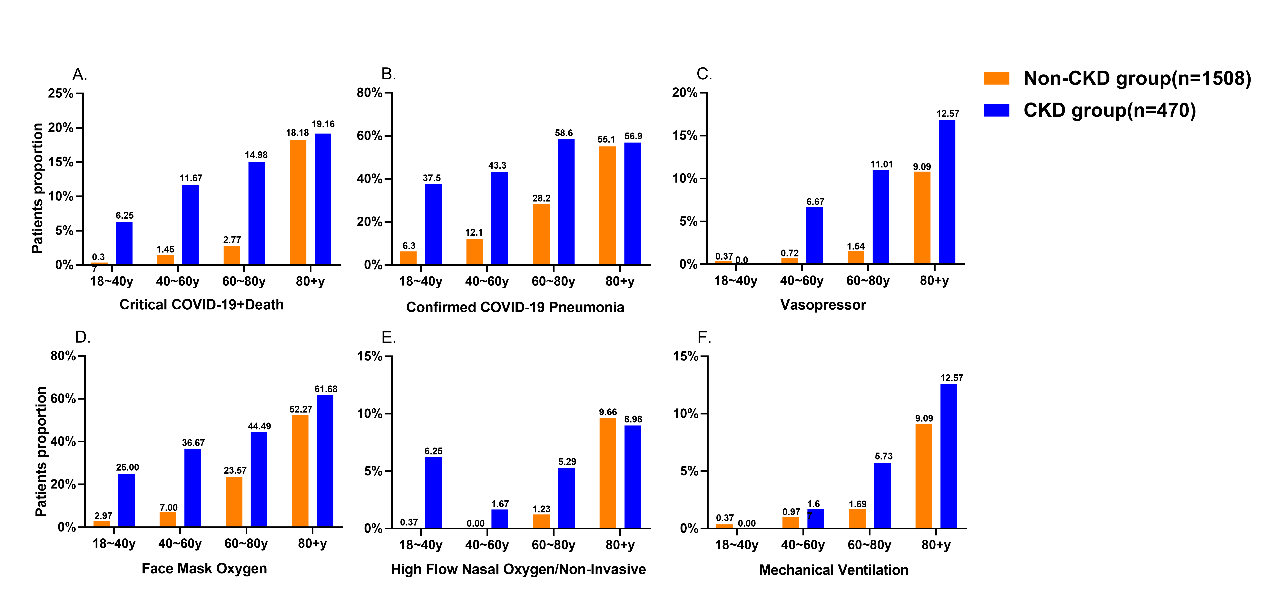

Supplement: Supplementary file 1 — Supplementary Material 1 [file 12879_2023_8620_MOESM1_ESM.docx]
